# Metastatic Breast Cancer Presenting As Neck Pain in a Pregnant Patient

**DOI:** 10.7759/cureus.51444

**Published:** 2024-01-01

**Authors:** Eric Chun-Pu Chu, Steve Ming Hei Yun

**Affiliations:** 1 Chiropractic and Physiotherapy Centre, New York Medical Group, Hong Kong, CHN

**Keywords:** chiropractor, chiropractic, neck pain, pregnancy, breast cancer

## Abstract

Breast cancer metastasizing to the spine during pregnancy is a rare occurrence. A 36-year-old woman experienced persistent neck pain during the third trimester of pregnancy. The pain continued until the postpartum period, severely affecting quality of life (QOL). Physical examination revealed a restricted cervical range of motion. Spinal lesions were detected on magnetic resonance imaging. Metastatic breast cancer was confirmed through a biopsy. The patient underwent radiation therapy for spinal metastasis, chemotherapy for breast cancer, and nonsteroidal anti-inflammatory drug therapy for pain. She was referred for chiropractic care and physical rehabilitation. After six weeks of collaborative treatment, the patient experienced significant pain relief and improvement in strength, mobility, and QOL. This case report highlights the need to evaluate unexplained symptoms in pregnant and postpartum women to exclude sinister pathologies. It demonstrates the need for a multidisciplinary approach that combines oncological, chiropractic, and rehabilitative care to optimize the outcomes in patients with complex presentations.

## Introduction

Breast cancer during pregnancy (BCP) is a rare occurrence estimated to complicate approximately 1 in 3,000 pregnancies. However, it remains the most common malignancy diagnosed during gestation [1]. The mean age of women diagnosed with BCP is 32-38 years [2]. BCP commonly spreads to the lungs, liver, brain, and skeletal system [3]. Spinal metastasis poses complex diagnostic and therapeutic challenges that are distinct from those in non-pregnant populations.

Hormonal changes during pregnancy lead to breast enlargement, vascularity, and nodularity, which can obstruct early tumor detection [4]. Additionally, routine mammographic screening is often deferred until after child delivery, thus delaying diagnosis. Increased breast density further hampers the radiographic identification of early-stage breast cancer in pregnant patients [5]. Consequently, breast cancer is often diagnosed at a more advanced stage in pregnant patients compared with that in age-matched controls [6].

This case report describes a 36-year-old woman with breast cancer metastasis to the cervical spine diagnosed postpartum who originally presented with neck pain during pregnancy. This underscores the need for a prompt workup of unexplained symptoms in pregnant and postpartum women to exclude sinister etiologies. This case report also highlights the importance of implementing a collaborative multidisciplinary approach in managing the complexities of BCP.

## Case presentation

A 36-year-old housewife presented to a chiropractic clinic with a five-month history of neck pain that began in the third trimester of pregnancy. The baby was delivered via cesarean section two months prior to the study. During the study, the infant was already one month old and exclusively breastfed. Her first child was aged two years, and she did not undergo breast cancer screening after giving birth to her first child. The patient experienced neck pain with an insidious onset and an intensity of 6 out of 10. The pain was localized to the right cervicothoracic region and radiated to the upper trapezius muscle without any accompanying numbness or tingling. The pain persisted throughout the day and was exacerbated by head rotation. Neck pain significantly affects a patient’s quality of life (QOL). She reported difficulties performing ordinary household chores and caring for her two children, especially the newborn. The patient frequently experienced sleep interruptions due to pain, which further affected her overall well-being and energy levels during the day. Her social interactions were also affected as the pain limited her ability to engage in activities outside the home. The overall QOL score was 60%.

During pregnancy and the postpartum period, the patient was unable to undergo radiography or extensive treatments for neck pain. She was diagnosed with pregnancy-related neck pain by an obstetrician and gynecologist and was only treated with non-steroidal anti-inflammatory drugs (NSAIDs). She sought traditional Chinese medicine, including neck manipulation and acupuncture, but did not experience any significant relief. She also underwent a conservative course of chiropractic care.

**Figure 1:**
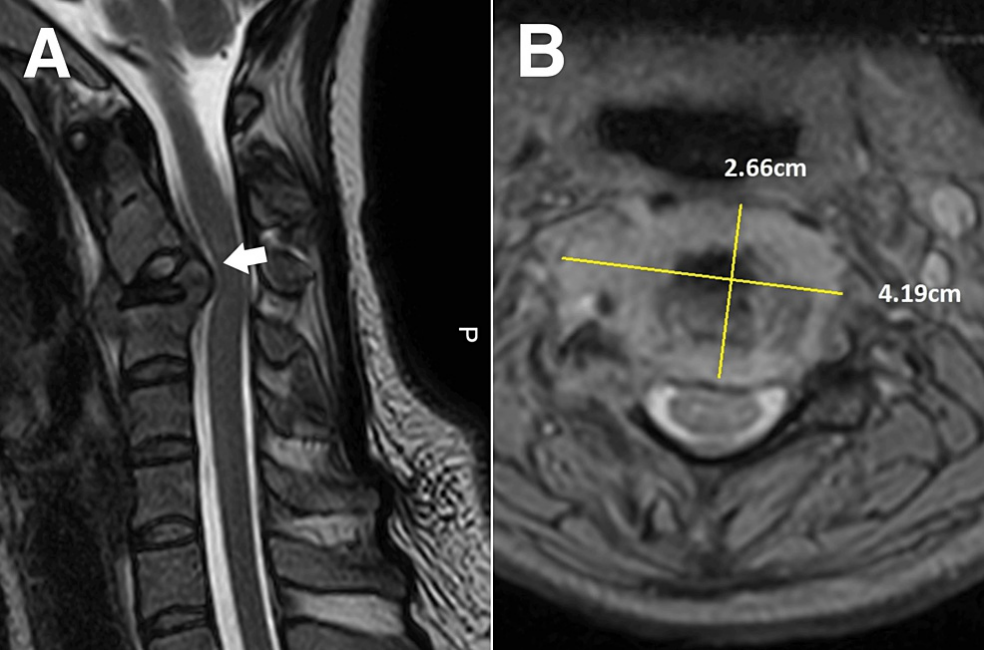
Cervical magnetic resonance image A) Vertebral plana in the C3 body with a T2 hyperintense lesion in C3. Mild cord compression at the C3 level. B) An overall size of 2.66 × 4.19 cm

During the chiropractic examination, the cervical range of motion was restricted as the pain radiated in all directions. Valsalva maneuver was performed, but no pain was elicited. However, cervical compression, Jackson compression, and cervical distraction were noted. The sensory and motor functions and reflexes were normal. Other tests, including the Kemp and heel-to-toe walk tests, yielded a negative result. Common conditions such as muscle strain, degenerative disc disease, and cervical spondylosis were considered in the differential diagnosis of persistent neck pain. However, considering that the patient's severe symptoms did not respond to conservative treatment and that the patient had no history of injury or structural arthritis to explain the symptoms, the chiropractor suspected a more serious underlying condition requiring further investigation. Given the patient's recent pregnancy and breastfeeding status, a magnetic resonance image (MRI) was arranged by the chiropractor to avoid exposure to ionizing radiation and diagnose early metastatic disease, infections, and inflammatory conditions. The radiologist reviewed the MRI scans and detected a vertebral plana at the C3 vertebral body (Figure 1A) and a T2 hyperintense lesion in C3, measuring 2.66 × 4.19 cm (Figure 1B). The radiologist informed the chiropractor that these findings were suggestive of metastasis. The patient was referred to an oncologist immediately.

**Figure 2:**
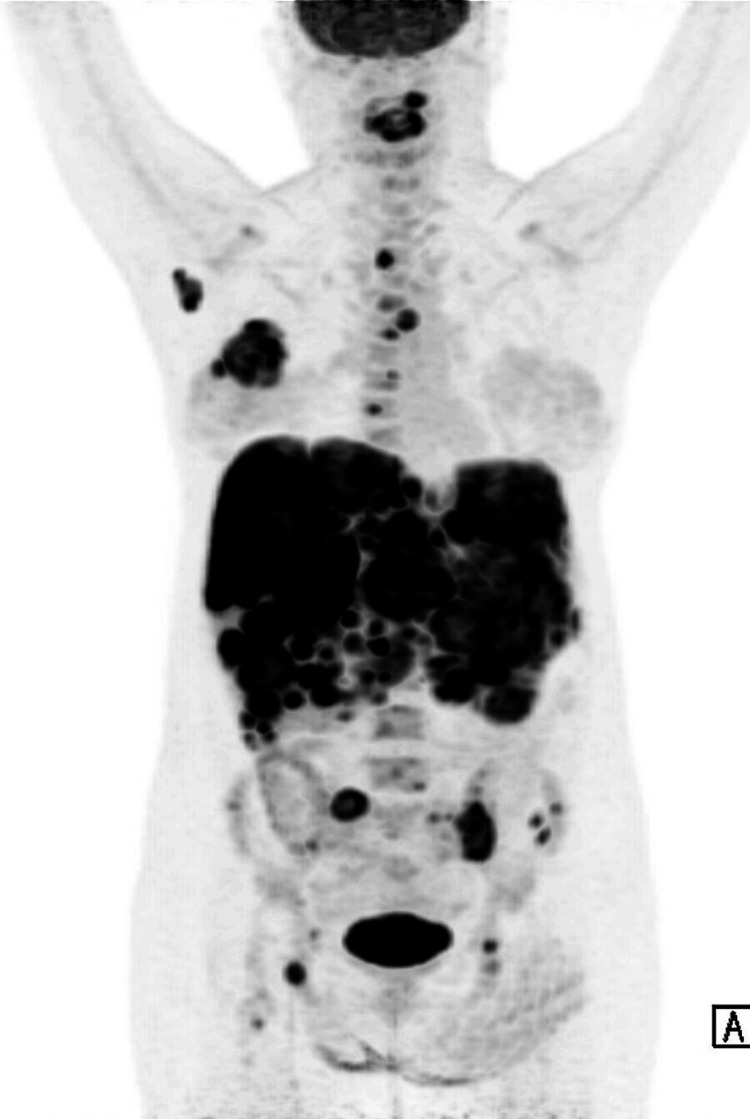
Positron emission tomography examination (PET) A hypermetabolic right breast mass is noted at 3 cm from the nipple. Multifocal liver metastases were noted diffusely infiltrating both hepatic lobes. Multiple focal bony metastases were noted in the axial and appendicular skeleton as described. Of note is the involvement of the cervical spine with vertebral plana deformity at C3 with bony retropulsion and compression of the spinal cord at C3/4 level.

The oncologist performed a detailed assessment and ordered a positron emission tomography examination (Figure 2) and biopsy. The biopsy confirmed the presence of malignant cells. Based on the radiological findings, biopsy results, and clinical presentation, a final diagnosis of metastatic breast cancer was made. A tailored treatment plan was initiated immediately. She underwent six weeks of radiation therapy targeting the metastatic cervical tumor. This was followed by hormonal therapy and chemotherapy for primary cancer. NSAIDs were also prescribed for mild to moderate pain.

Given the patient’s recent pregnancy and the potential side effects of cancer treatments, such as fatigue and weakness, the oncologist coordinated with the chiropractor to improve the patient’s physical function and QOL. The chiropractor worked closely with the oncologist, while the rest of the healthcare team designed a personalized rehabilitation program for the patient. The primary goals of the patient’s care were to alleviate pain, maintain neck mobility, and improve the overall physical function and QOL. The patient initially performed gentle range-of-motion exercises for the neck and upper body under the guidance of a chiropractor. Low-force spinal manual techniques, including activator and spinal mobilizations, were applied at the C5-T2 region. As the patient’s strength increased, more advanced exercises were introduced to improve muscle tone, balance, and endurance. The chiropractor also suggested various energy conservation techniques and safe methods to perform daily activities to help the patient better manage her energy levels and avoid exacerbation of neck pain.

The patient underwent six sessions of chiropractic care within a period of six weeks. As the treatment progressed, she reported significant pain relief, with improved strength and mobility. Her QOL score improved to 90%. Consequently, the patient was able to care for her children and participate in social activities.

## Discussion

Breast cancer metastasis to the spine causes spinal cord compression, with back pain as the cardinal presenting symptom [7-9]. However, in rare cases, metastases may manifest with atypical symptoms such as neck pain, as in the current patient. Although lower back pain has been frequently documented in both uncomplicated breast cancer [7-9] and BCP [10], its presentation as cervical pain is less commonly reported. This situation underscores the need for vigilance even if the symptoms do not coincide with the classic clinical picture. Persistent or unexplained musculoskeletal complaints in pregnant or postpartum women should prompt consideration for advanced imaging to exclude sinister etiologies such as metastatic disease, despite the nonspecific findings obtained during examination [10]. Maintaining a low threshold for imaging is especially prudent considering that spinal involvement portends morbidity from nerve compression. Thus, the unusual manifestations of common serious conditions should not be overlooked in vulnerable populations.

This case report also reinforces the idea that chiropractors should recognize subtle warning signs and maintain a broad differential diagnosis, including breast cancer when assessing patients with musculoskeletal pain [10]. MRI can be used as a diagnostic tool for conducting high-risk screening for a diverse range of breast abnormalities, including those specific to pregnancy and breastfeeding [4]. These abnormalities include physiological alterations, benign illnesses, and malignant neoplasms [4]. When the MRI of the present patient showed spinal lesions, the chiropractor made a crucial decision to refer the patient for prompt oncological evaluation and care. This situation underscores the importance of communication and collaboration between chiropractors and healthcare providers from other disciplines, such as oncology and radiology when clinical suspicion arises.

Although not involved in cancer treatment, the chiropractor plays a pivotal role in the patient’s multidisciplinary care team. The chiropractor helped optimize pain relief, spinal stability, mobility, and functional status throughout the course of patient care [7-9].

This study provides two key insights. First, it demonstrates the role of chiropractors in identifying subtle warning signs to prompt advanced imaging and referral when appropriate. Second, it highlights the importance of chiropractic care in a multidisciplinary approach to managing complex conditions by providing complementary pain and mobility support.

## Conclusions

This case report describes an uncommon presentation of breast cancer metastasis to the cervical spine in a pregnant patient that initially manifested as neck pain. The insidious onset of severe persistent pain warrants an MRI, which reveals the presence of spinal lesions. Prompt referral to the Oncology Department led to a postpartum diagnosis of metastatic breast cancer. This case highlights the need to maintain a low threshold for advanced imaging when unexplained musculoskeletal symptoms arise in pregnant and postpartum women, even in the absence of significant objective findings. It also demonstrates the merit of a collaborative multidisciplinary approach, with the chiropractor contributing to vital pain management and physical rehabilitation alongside primary oncological treatment. Although this report adds to the limited literature on the atypical presentations of breast cancer metastasis in pregnancy, the findings may not be generalizable owing to the individual complexity of this case. Overall, the key insights are the role of clinical judgment in guiding appropriate imaging to exclude sinister pathology and the value of coordinated care among healthcare providers from various specialties such as chiropractic, oncology, and radiology in optimizing patient outcomes when navigating diagnoses such as cancer during pregnancy. Further research is warranted to determine ideal diagnostic protocols and the need for the integration of supportive care in these complex scenarios.
